# Why does my patient’s basilar artery continue to grow? A four-year case study of a patient with symptoms of vertebrobasilar dolichoectasia

**DOI:** 10.1186/s12883-018-1045-0

**Published:** 2018-04-20

**Authors:** Dao Pei Zhang, Yan Fang Peng, Qian Kun Ma, Min Zhao, Huai Liang Zhang, Suo Yin

**Affiliations:** 1 Department of Neurology, The First Affiliated Hospital of Henan University of CM, Zhengzhou, China; 2 Department of Neurology, People’s Hospital of Zhengzhou Affiliated to Southern Medical University, Zhengzhou, China; 3 Department of Neurology, People’s Hospital of Henan Province, Zhengzhou, China; 4 Department of Image, People’s Hospital of Zhengzhou Affiliated to Southern Medical University, Zhengzhou, China

**Keywords:** Vertebrobasilar dolichoectasia, Obstructive hydrocephalus, Stroke, Artery dissection, Magnetic resonance imaging

## Abstract

**Background:**

Vertebrobasilar dolichoectasia (VBD) is a clinical entity associated with ischemic stroke, compression of cranial nerves or brainstem, and hydrocephalus. There have been relatively few studies following the progression of VBD in patients presenting with a variety of diverse clinical features.

**Case presentation:**

Here, we report a case study of a male with progressive VBD who was followed from November 2012 to December 2016. The patient had diagnosed hypertension for several years and suffered from left peripheral facial paralysis, recurrent ischemic attacks in the brainstem and cerebellum, obstructive hydrocephalus and frequent pneumonia. A series of cranial CT and multi-modal MRI scans were performed to explore the brain imaging features of the patient during follow-up.

**Conclusions:**

The presented case study suggests that aging, uncontrolled hypertension, arterial dissection and infection may contribute to the exacerbation of VBD and recurrent ischemic stroke.

## Background

Vertebrobasilar dolichoectasia (VBD) is a rare disease characterized by expansion, elongation, and tortuosity of the vertebrobasilar arteries [1]. Though the prevalence of VBD has been estimated to be in the range of 0.2–4.4% for the general population and 2.6–17.1% for stroke patients [2], reliable population-based data are lacking. Complications from VBD include transient ischemic attack, compression of brainstem or cranial nerves, ischemic stroke, subarachnoid hemorrhage, and obstructive hydrocephalus [1, 3]. A previous systematic review of 375 patients concluded that patients with VBD are at an elevated risk of neurological deterioration during a 5-year follow-up [2].

Generally, VBD has is considered to be a progressive clinical entity that leads to heightened morbidity and mortality [4]. However, few studies have included patients exhibiting diverse clinical features with an elaborate evolving process of VBD. Furthermore, the factors that contribute to VBD growth remain unclear.

The current paper reports the case of a 52-year old man with hypertension who suffered from a series of symptoms over four years, including peripheral facial paralysis, recurrent ischemic stroke attacks affecting subtentorial areas, and obstructive hydrocephalus. Importantly, the patient presented with a gradually growing VBD, slow blood flow, basilar artery (BA) vessel wall hematoma, and vertebral dissection revealed by multi-modal magnetic resonance imaging (MRI). Here we describe VBD progression and growth and discuss possible contributing factors.

## Case presentation

**Fig. 1:**
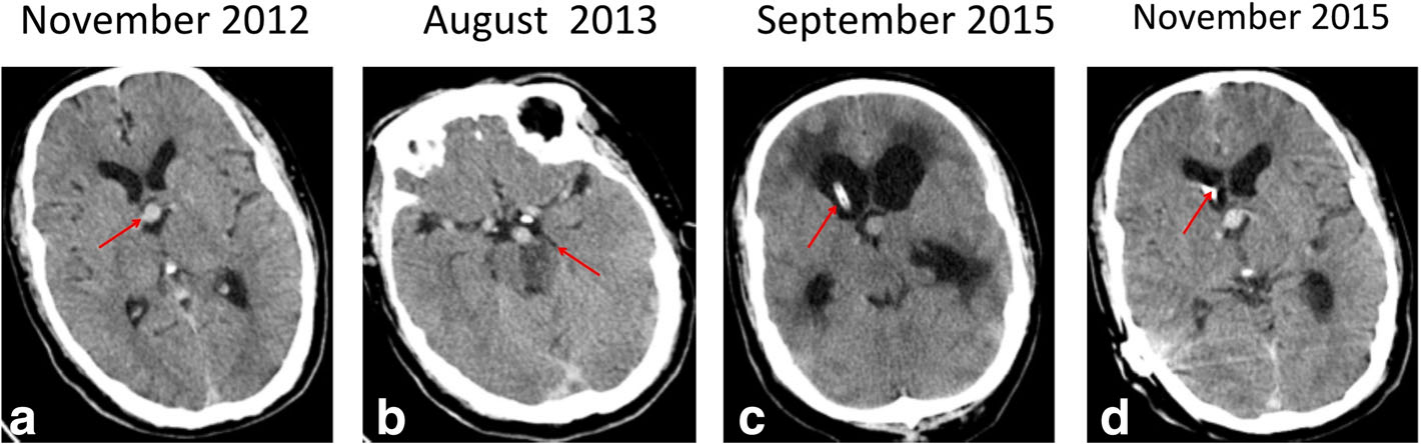
Serial axial non-enhanced CT scans. (**a**) Upward displacement of the third ventricle (red arrow) by a markedly ectatic BA. (**b**) Left pontine infarction compressed by the ectatic BA (red arrow). (**c**) Bilateral dilated ventricles and low density lesions anterior and around the lower horns on the second day after ventriculoperitoneal shunt placement (red arrow). (**d**) Reduced ventricular dilation and disappearance of low density lesions around the ventricle horn (red arrow) one month after shunt placement

The presented patient developed left side peripheral facial paralysis in November 2012. A cranial computed tomography (CT) scan showed an elongated and dilated BA, of high density without contrast enhancement, that passed upward to enter the floor of the third ventricle (Fig. 1a). A head CT angiography later verified a diagnosis of VBD (CT imaging data are not available).

In August 2013, the patient was hospitalized for three days due to dizziness, weakness and numbness of the left limbs, and alalia. Neurological examinations revealed loss of muscle strength [grade 3 on the Medical Research Council (MRC) scale] and moderate loss of pinprick and temperature sensation in the left limbs without involvement of the face. A CT scan showed a right pontine infarction and the same elongated BA that was seen in November 2012 (Fig. 1b). The patient declined multi-modal MRI.

**Fig. 2:**
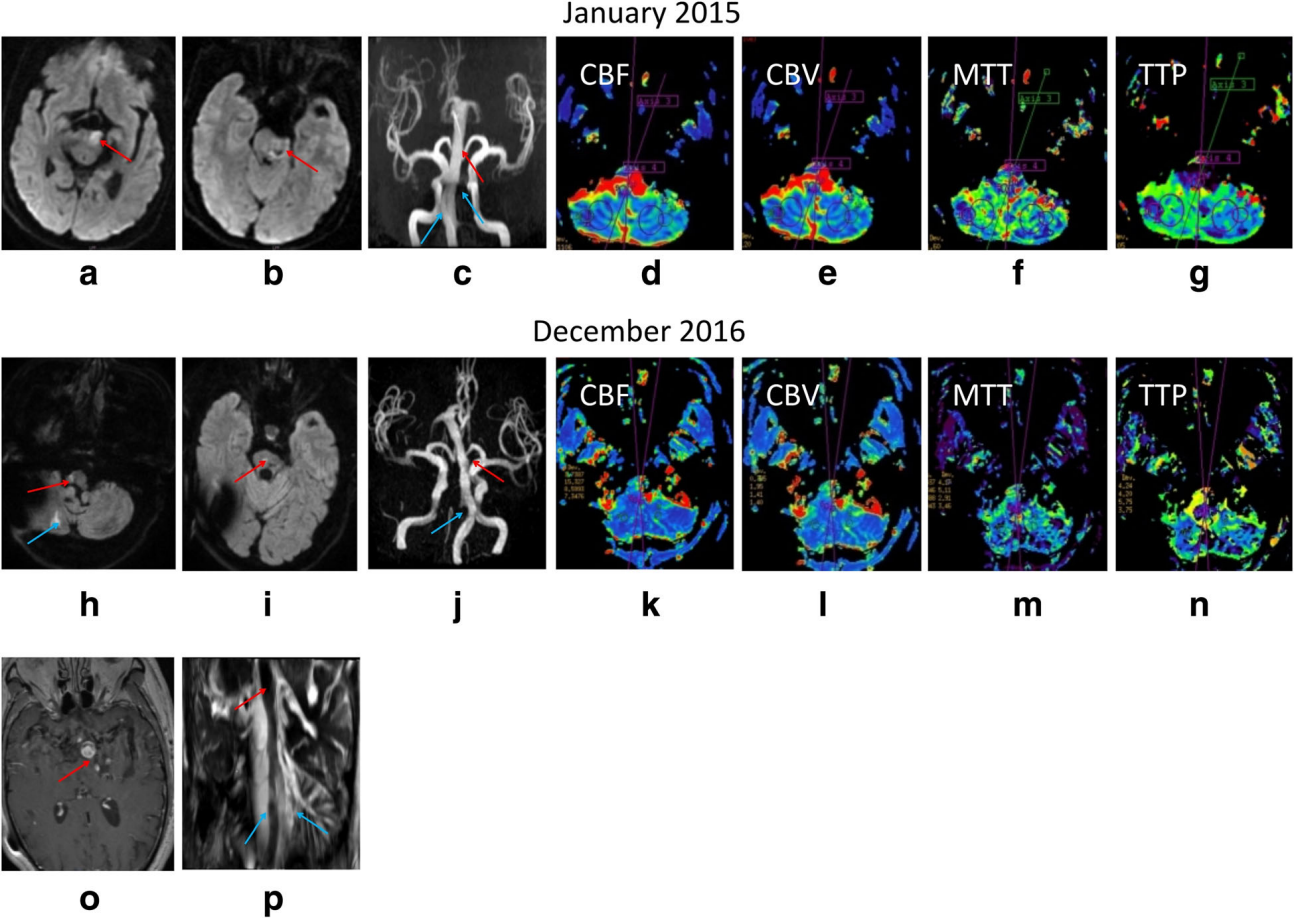
Follow-up multi-modal MRI scans. (**a**) DWI showing acute ischemic infarction in the left mesencephalon-pontine junction (red arrow). (**b**) DWI showing old infarction with reactive gliosis (red arrow). (**c**) TOF-MRA demonstrating an abnormal BA caliber (red arrow), bifurcation at the third ventricle floor, and bilateral vertebral arteries are present with enlarged diameters (blue arrow). (**d**-**g**) PWI showing decreased cerebral blood volume (CBV) and cerebral blood flow (CBF) of the medulla oblongata, pons, and cerebellum, and elongated MTT and TTP of the right cerebellum. (**h**) DWI showing acute ischemic infarction in the right medulla oblongata (red arrow) and cerebellum (blue arrow). (**i**) DWI showing acute ischemic infarction in the right pons with Wallerian degeneration (red arrow). (**j**) TOF-MRA showing an enlarged diameter and increased length of the BA (red arrow), bifurcation at the level of the bilateral ventricles, and non-observable right vertebral artery (blue arrow). (**k**-**n**) PWI shows decreased CBV and CBF of the bilateral medulla oblongata, and cerebellum, and elongated TTP of the right medulla oblongata. (**o**) High-resolution MRI showing signs of a double lumen of the BA (red arrow). (**p**) Basi-parallel anatomic scanning showing a larger diameter of the BA than that seen with TOF-MRA (red arrow) and an observable right vertebral artery (blue arrow)

The patient was again hospitalized for two days in January 2015 due to dizziness and numbness of the right limbs with bucking and alalia. Neurological examination revealed dysarthria, dysphagia, severe right hemiplegia, with muscle strength of the upper limb at a grade 2 and the lower limb at a grade 3 (MRC) and loss of pinprick sensation in the right limbs. Diffusion-weighted imaging (DWI) showed acute ischemic infarction in the mesencephalon-pontine junction (Fig. 2a and b). Three-dimensional time-of-flight MR angiography imaging (TOF-MRA) revealed a dilated, tortuous, and elongated vertebral artery and BA (Fig. 2c). Dynamic susceptibility contrast-enhanced perfusion weighted imaging (DSC-PWI) showed decreased cerebral blood volume (CBV) and cerebral blood flow (CBF) in the medulla oblongata, cerebellum, and pons, and elongated mean transition time (MTT), and time to peak (TTP) in the left cerebellum, revealing multiple relatively ischemic territories of the posterior circulation (Fig. 2d-g).

**Fig. 3:**
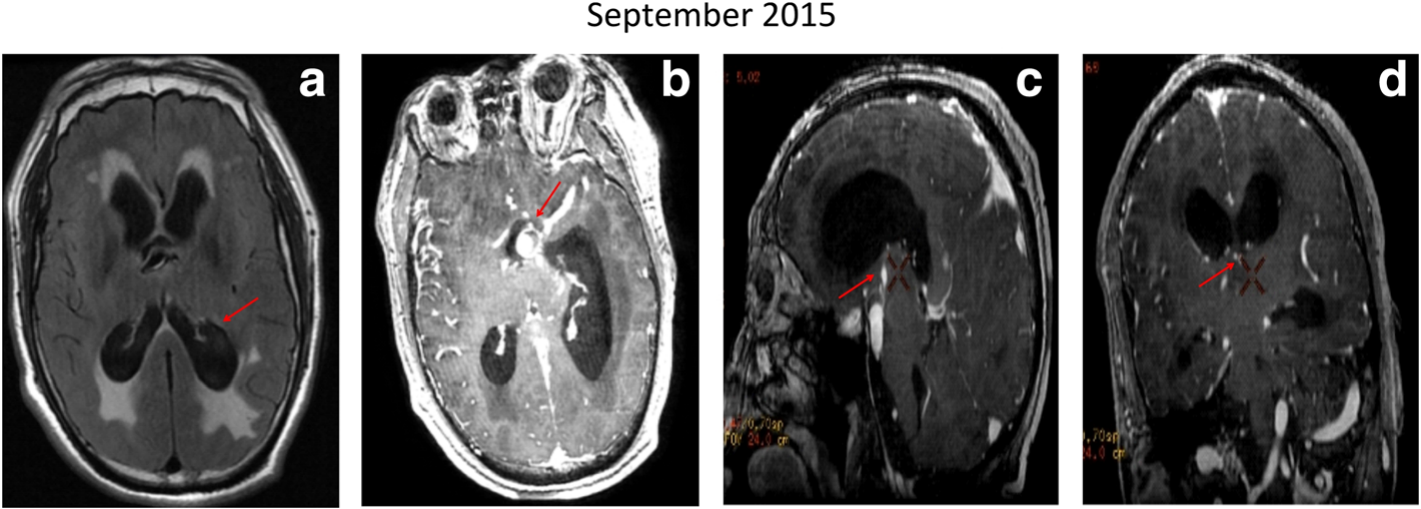
Non-enhanced and contrast-enhanced MRI scans. (**a**) Axial T1-weighted image showing a dilated lateral ventricle and encircled calyptriform with high intensity (red arrow). (**b**) Enhanced axial T1-weighted image showing BA vessel wall hematoma (hyperintensities and double lumen, red arrow). (**c**) Enhanced sagittal T1-weighted image showing bifurcation toward the base of the lateral ventricle (red arrow), a narrowed aqueduct of sylvius, a distorted third ventricle, and dilation of the lateral ventricle. (**d**) Enhanced coronal T1-weighted image revealing compression of the foramen of Monro due to VBD

In September 2015, the patient’s symptoms worsened, and he was again hospitalized in a somnolent state with a Glasgow Coma Scale score of 12 (eye opening score = 3, verbal response score = 4, and motor response score = 5). Neurological examination showed bilateral paralysis with MRC grade 0, grade 3, and grade 4 muscle strength of the right limbs, left upper limb, and left lower limb, respectively. Non-enhanced and contrast-enhanced MRI and MRA showed obstructive hydrocephalus due to the growth of the BA (with a double lumen sign suggesting dissection), resulting in the compression of the foramen of Monro (Fig. 3a-d). An emergency ventriculoperitoneal shunt surgery was performed. One month later, reexamination by CT demonstrated that the hydrocephalus had decreased (Fig. 1c-d). The patient was discharged from the hospital after exhibiting recovery of his limb muscle strength during his hospital stay, at which time he still required supportive care in his daily life.

In December 2016, the patient was wheel chair-bound and complained of dizziness and dysphagia. He was thus hospitalized again. Neurological examinations revealed hypoactive pharyngeal reflex, dysphagia, dysarthria, internuclear ophthalmoplegia, ataxia and paralysis of the right upper and lower limbs. Acute multiple spot-like infarctions (artery-to-artery embolism) involving the left medulla oblongata, cerebellar hemisphere and pons were found through DWI (Fig. 2h and i). In addition, the right vertebral artery was not visible (Fig. 2j). High-resolution MRI showed a double lumen of the BA (Fig. 2o). Compared to TOF-MRA imaging, basi-parallel anatomic scanning [5] showed a greater BA diameter and a visible right vertebral artery, suggesting dissection (Fig. 2p). PWI revealed hypo-perfusion of the medulla oblongata, cerebellar hemispheres, and pons (Fig. 2k-n). After approximately 20 days of antiplatelet therapy, the patient was discharged in a severely disabled condition characterized by bed-bound paralysis with MRC grade 1 muscle strength of the right upper limb and both lower limbs, requiring continuous care and attention.

**Fig. 4:**
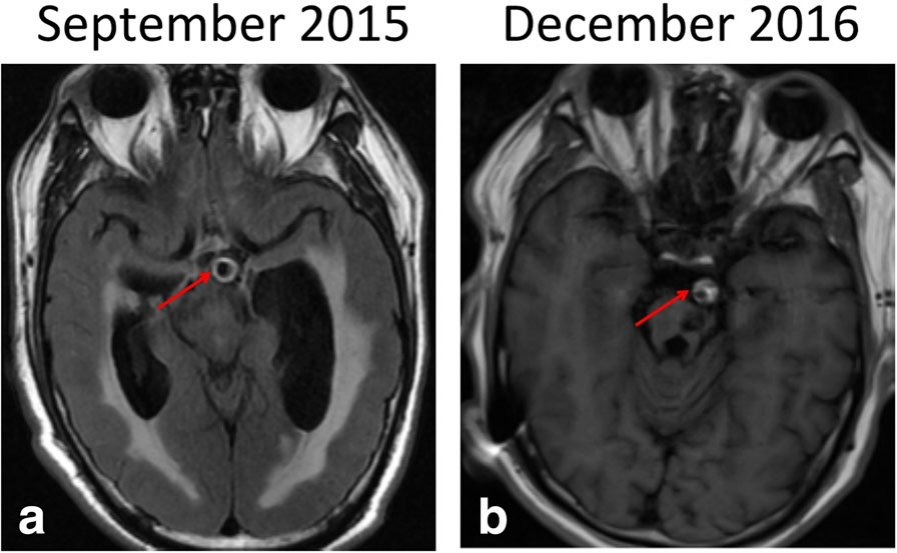
MRI-FLAIR scans. (**a**) A hyperintense rim near the vessel wall (FVH grade 2). (**b**) A hyperintense signal that nearly fills the entire BA lumen (FVH grade 3)

**Fig. 5:**
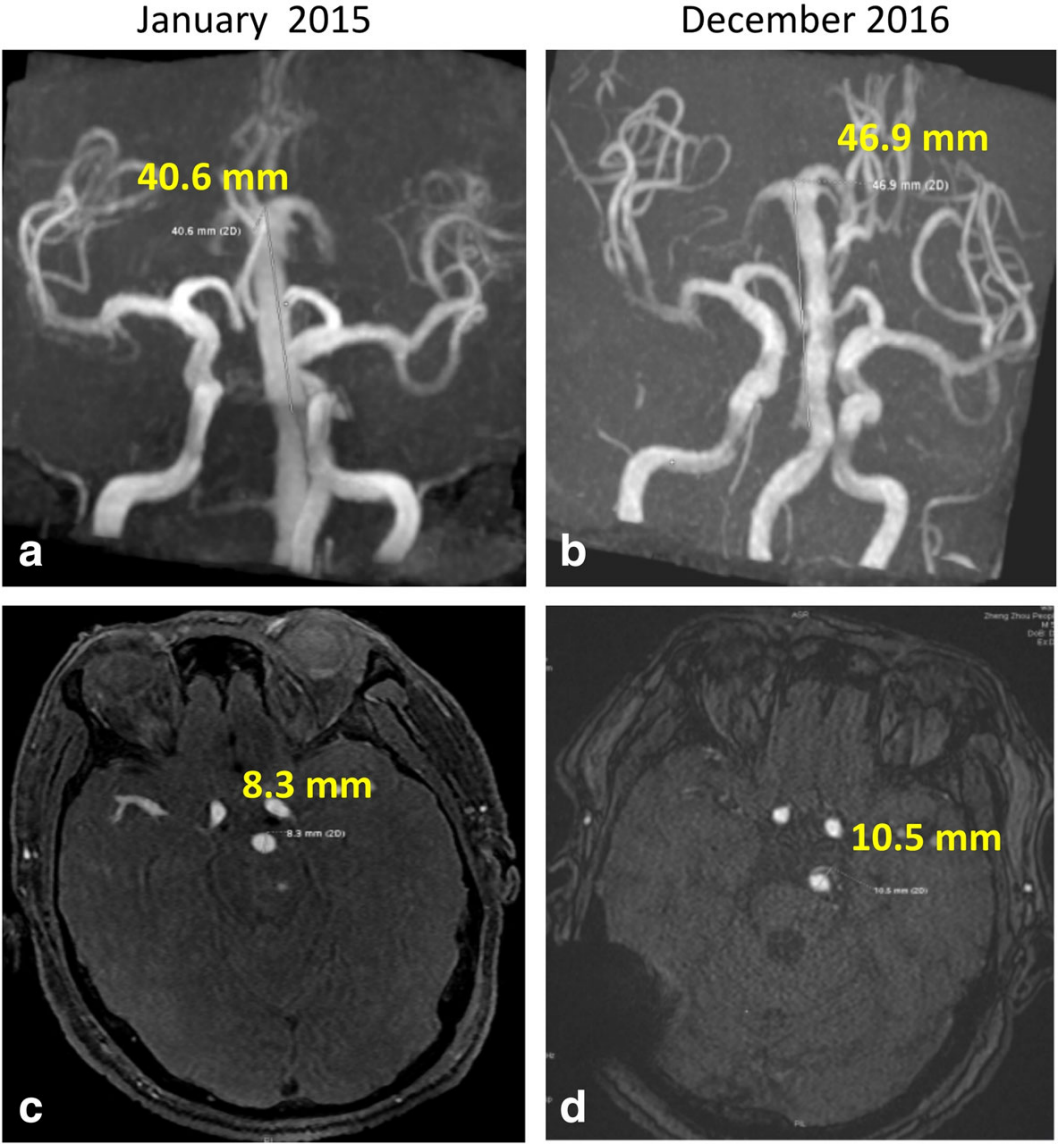
TOF-MRA scans with BA growth images. (**a**, **c**) TOF-MRA images from January 2015 revealing a tortuous and elongated BA (**a**: length, 40.6 mm) with dilation (**c**: diameter, 8.3 mm). (**b**, **d**) TOF-MRA images from December 2016 revealing increases in BA tortuosity, elongation (**b**: length, 46.9 mm) and dilation (**d**: diameter, 10.5 mm)

In September 2015 and December 2016, fluid attenuated inversion recovery (FLAIR) vascular hyperintensity (FVH 3) [6] around the rim of the BA wall indicated slow blood velocity (Fig. 4a and b). In January 2015, TOF-MRA revealed a tortuous, elongated (length, 40.6 mm), and dilated (diameter, 8.3 mm) BA. In December 2016, TOF-MRA revealed greater tortuosity, further elongation (length, 46.9 mm), and further dilation (diameter, 10.5 mm) of the BA (Fig. 5a-d). Unfortunately, in March 2017, the patient died of pneumonia secondary to recurrent aspiration due to lower cranial nerve deficits.

## Discussion and conclusions

The diagnosis of BA ectasia is defined by dilation reaching > 4.5 mm in diameter at the mid-pons level [7]. An increase in BA diameter by ≥2 mm from the baseline diameter or an increase in lateral displacement or height of BA bifurcation is classified as a progression of VBD [8]. In this case, serial TOF-MRA studies conducted almost 2 years apart (January 2015 to December 2016) revealed increases in BA tortuosity, elongation (40.6 mm to 46.9 mm), and dilation (8.3 mm to 10.5 mm). BA dolichoectasia rupture risk has been shown to be associated with an initial artery diameter larger than 10 mm [9]. Alternative treatments, such as blood pressure reduction therapy, surgery, or endovascular procedures, may be considered in cases of BA enlargement (BA diameter ≥ 10 mm) [9]. It is of critical importance to follow the development and progression of patients with VBD so as to implement a proper and timely intervention to improve the patient’s prognosis [10].

A recent case report followed a 60-year-old Caucasian man with a 31-year history of secondary progressive multiple sclerosis and uncontrolled arterial hypertension. The report presented a series of images over many years demonstrating the progression of a normal BA becoming dolichoectatic, followed by the formation of an aneurysm [11]. Here, we present a four-year follow-up case of a male with unilateral facial paralysis that was indefinitely associated with VBD, who showed gradual development of recurrent ischemic stroke and obstructive hydrocephalus. The maximum diameter and length of his BA, as revealed by MRA, increased gradually. Moreover, the lateral height of BA bifurcation was increased from the level of the interpeduncular cistern or the suprasellar cistern to the level of the third and lateral ventricles.

The normal blood flow velocity profile over a longitudinal section is parabolic with lowest velocities typically observed near the vessel wall. The blood flow in a patient with VBD is turbulent and slow, causing hypoperfusion, as displayed on PWI [10]. Perfusion images in the current case study, showed hypoperfusion in the blood supply area of VBD. Moreover, a prior study indicated that FVH may demonstrate decreased blood flow velocity in patients with VBD [9]. The presence and extent of FVH in the BA is classified into three grades and the current patient reached grade 3, at which time the hyper-intense signal filled the entire lumen. Reduced blood flow can cause hypoperfusion, leading to ischemic symptoms. The recurrent strokes experienced by the presented patient may be partially associated with abnormal VBD hemodynamics. The patient’s latest spot-like infarction might have been caused by artery-to-artery embolism and hypoperfusion. However, progressive enlargement of the BA may also pull or compress the origin of the perforating artery, leading to infarction of the blood supply territory [9]. Because DWI showed acute multiple spot-like infarctions involving the left medulla oblongata, cerebellar hemisphere, and pons, the latter mechanism was ruled out in our patient.

Although VBD-associated recurrent ischemic events were observed, the plan for secondary prevention was poor [12]. The general inadequacy of the literature related to VBD-associated stroke treatment do not provide an avenue for high-confidence treatment planning based on guidelines [2]. Aspirin and anticoagulants are an option, but would increase intracerebral bleeding risk [13]. Therefore, we pursued cautious antiplatelet therapy in this case. The efficacy of surgical and radiological interventions should be determined in future studies.

Imaging data confirmed a BA vessel-wall hematoma and dissection of the right vertebral artery. Vertebrobasilar artery dissection may contribute to the deterioration of symptomatic VBD. Furthermore, based on findings indicating that VBD is related to arterial dissection and the presence of a vessel wall hematoma, the authors of a previous study suggested that the two pathologies may share etiologies [14]. Additionally, abnormal hemodynamic stress may play an important role in arterial dissection and vessel wall hematoma formation [15, 16]. Thus, vertebrobasilar artery dissection may accelerate the progression of VBD [15]. Importantly, vessel wall hematoma development is also a crucial step in the pathological progression of dolichoectasia [17]. In the current case study, the patient had developed a BA vessel-wall hematoma and vertebral artery dissection with concurrent obstructive hydrocephalus and severe ischemic strokes. MRA revealed a dilated BA that extended towards the third ventricle. It may be speculated, therefore, that the development of obstructive hydrocephalus was associated with compression of the foramen of Monro, the cerebral aqueduct, or the third ventricle [18].

The reason for the rapid progression of VBD in the current case is unknown. It is possible that increased blood pressure may have aggravated the natural course of VBD [19]. Hypertension can increase the wall shearing stress of a dolichoectatic BA, leading to the formation of atherosclerosis and, ultimately, to cerebral ischemic stroke. In the presented case, the patient had developed frequent pneumonia due to recurrent posterior circulation ischemic stroke. The occurrence and growth of VBD has been attributed to the interaction between multiple factors, including infection and immune status [1]. A recent study noted that HIV infection was associated with artery adventitial inflammation and dolichoectasia independent of intracranial atherosclerosis, suggesting that differential inflammatory responses may influence the clinical course of intracranial atherosclerosis and dolichoectasia [20]. The post-stroke inflammatory response is the predominant mechanism linking ischemic stroke with infection. In turn, infection can promote atherosclerosis, plaque rupture, and thrombosis, leading to ischemic stroke [21]. Atherosclerosis is often associated with VBD, and may be a pathogenic factor of VBD. An autopsy of a deceased patient who had been diagnosed with an IgG4-related disease revealed some histological features of vertebral arteries and BAs that were very similar to those of an inflammatory aortic aneurysm, suggesting that autoimmune and inflammation mechanisms may also play a role in the etiology of VBD [22]. Therefore, we hypothesize that inflammatory cytokines may promote the fracture or destruction of the inner elastic layer of vessel walls and hyperplasia of intima-media smooth muscle cells, leading to further BA ectasia.

In conclusion, the current case study presents the clinical course of a patient who experienced the full spectrum of VBD symptoms within a four-year period. The clinical course, along with a series of CT and MRI studies, demonstrates the evolving process of VBD. Uncontrolled hypertension, repeated infections, and arterial dissection all contributed to the advancement of VBD. Future research should focus on the progression of VBD through multiple modality imaging to gain insight into the underlying pathophysiologic mechanisms of this disease.

## Abbreviations

BA: basilar artery
CBF: cerebral blood flow
CBV: cerebral blood volume
CT: computed tomography
DSC-PWI: dynamic susceptibility contrast-enhanced perfusion weighted imaging
DWI: diffusion-weighted imaging
FLAIR: fluid attenuated inversion recovery
FVH: flair vascular hyperintensity
MRC: Medical Research Council
MRI: magnetic resonance imaging
MTT: mean transition time
TOF-MRA: time-of-flight MR angiography imaging
TTP: time to peak
VBD: vertebrobasilar dolichoectasia
